# Editorial: Multiplexed image analysis for translational research project applications

**DOI:** 10.3389/fonc.2022.1089265

**Published:** 2022-11-17

**Authors:** Vera Luiza Capelozzi, Edwin Roger Parra

**Affiliations:** ^1^ Department of Pathology, The University of São Paulo Medical School, São Paulo, Brazil; ^2^ Department of Translational Molecular Pathology, The University of Texas MD Anderson Cancer Center, Houston, TX, United States

**Keywords:** image analysis, multiplexed immunohistochemistry/immunofluorescence (mIHC/IF), translational studies, immune profiling, tumor tissues

With the continuous improvement in our understanding of immuno-oncology and in the therapeutic arsenal for establishing personalized treatment for patients, tumor biomarkers targeting specific disease patterns are necessary. The evaluation of protein expression employing immunostaining of formalin-fixed, paraffin-embedded (FFPE) specimens is an ancillary technique for guiding clinical decisions in multiple situations, such as tumor stratification, evaluation of disease recurrence, precise mutation discovery, and subsequent targeting of treatment towards specific biomarkers. However, standard enzyme-based chromogenic immunohistochemistry (IHC) is a classic technology to study tumor microenvironments has limitations such as the use of separate slides per marker or challenges to analyze markers that are expressed by different cell populations with digital image analysis (1). In contrast to standard bright-field IHC staining, multiplexed image analysis technologies offer the opportunity to observe several markers independently in one tissue section without staining each marker on separate slides (2). Multiplexed analysis also allows accurate protein co-localization, stain adjustment, objective scoring, and identification of cut-off values for each biomarker. These properties are critical in low immune-stained areas, which represent difficult areas to evaluate on immunostained glass slides and may be misinterpreted as unfavorable because of camouflaging by the hematoxylin counterstain (3). In this Research Topic, we highlight a series of publications focusing on the applicability of the commercial modalities in most common use multiplexed imaging modalities to enable prospective users to conduct translational research and tumor immune environment characterization.

Several highly multiplexed tissue imaging technologies have also emerged involving cyclic immunofluorescence (IF), tyramide‐based IHC/IF, epitope‐targeted mass spectrometry, or RNA detection, allowing detailed studies of cell structure, functional properties, and cell–matrix cross-talk, facilitating diagnosis and targeted therapy. Coupling such advanced technologies with multiplexed image investigation can enable estimation of the cellular spatial arrangement, permitting a better understanding of carcinogenesis and sensitivity to immunotherapy. However, routine implementation of these technologies still depends on method standardization. In this context, multiple efforts to establish guidelines of reproducibility for use of these technologies in research and clinical applications are under way: by several task forces, including the Society for Immunotherapy of Cancer (SITC) (4); working groups, including the Joint Effort to Develop multiplex Immunofluorescence standards (JEDI) council (5); and multi-institutional studies, such as the Multiplexed Immunofluorescence Reproducibility Evaluation (MITRE) study (6) and the Cancer Immune Monitoring and Analysis Centers–Cancer Immunologic Data Commons (CIMAC-CIDC) network (7). Multiplexed image technologies can provide much information to drive new approaches for personalized medicine for immunotherapy when the samples are limited for different studies during clinical trials or diagnosis. It’s worth emphasizing this advantage when the diagnostic tissue specimen is a limiting factor. Most notably, panel of biomarkers can be selected to characterize tumor tissues and their localization in these specimens using multiplexed image technologies (8). It has been a consensus among pathologists that standard pathology analyses using bright-field microscopy remain the primary tools for diagnosing some pathologies (9). However, such technique has significant restrictions, including selection bias, and subjective interpretation.

To address these restrictions, Hristu et al. used two nonlinear optical contrast modalities—two-photon excited fluorescence (e.g., elastic fibers, keratin, lipofuscin, or melanin pigment) and second harmonic generation (e.g., collagen and myosin)— to concurrently evaluate Paget disease on the breast skin using FFPE specimen sections stained by IHC markers and hematoxylin and eosin. They found that two-photon excited fluorescence distinguished particular cell characteristics of Paget disease on the breast skin, equivalent to histopathological findings.

As well as two-photon microscopy to evaluate in-depth-phenotyped cells, imaging mass cytometry (IMC), another multiplexed image technology, permits the *in-situ* analysis of individual cells in the innate microenvironment of normal and diseased tissues within the preserved histoarchitecture of a unique specimen section. Thus far, the IMC technology allows the concurrent evaluation of about 50 antibodies’ expression by a metal conjugation kit that labels antibodies or proteins. Le Rochais et al. critically analyzed articles on using IMC technology in malignant tumors and described the IMC method for deciphering the heterogeneous tumor microenvironment (TME) field. The application of IMC is extensively discussed in cancer research as a means of defining predictive and prognostic biomarkers and facilitating our comprehension of how cancer spreads and overcomes immune system defenses.

To characterize the TME through immunoprofiling and establish prognostic and predictive markers of immunotherapy response is essential, and multiplexed technologies can help. Hernandez et al. performed a critical literature review of studies using the nanoString GeoMx^®^ Digital Spatial Profiler (DSP). This platform permits high-plex protein and mRNA expression profiling, providing spatial and temporal characterization of frozen and FFPE tumor specimens. These authors discussed relevant studies showing the DSP technology’s feasibility for finding predictive biomarkers for immunotherapy in different tumor types. In addition, the DSP was shown to more accurately investigate many biomarkers compared to similar high-technology platforms.

To characterize the TME and to explore the applicability of mIHC/IF in routine practice, a literature review by Rojas et al. suggests that pathology laboratories will have the crucial mission of implementing multiplexed technology and validate these technologies by conducting retrospective analyses of clinical trial samples and creating reproducible reports, to establish uniform guidelines and pipelines for mIHC/IF. In addition, Rojas et al. discuss the necessity of using mIHC/IF for clinical practice and of proposed assay harmonization. Translating mIHC/IF so that it facilitates medical decisions is vital, considering cost/benefit concerns in immunotherapy; implementing diagnostic testing that accurately previews effects can minimize expensive immuno-oncology treatment.

Sundaram et al. demonstrated the importance of inflammatory environment changes and their spatial interactions in the epithelial transformation to esophagus adenocarcinoma, using a sequential mIHC platform. They analyzed 10 immune cell biomarkers simultaneously and showed their importance in this disease. Moreover, they identified a previously unknown lineage of myeloid cells that increased with late disease stage. Interestingly, these authors draw attention to the importance of multiplex imaging equipment, combined with semi-automated image analysis using FFPE sections, to examine the immune landscape in the tumor biome.

Highlighting the importance of computational image learning and an algorithm for training feedforward neural networks in multi-omics analyses and multiplexed image technologies, Tu et al. examined the role of the FAM83A gene in the process of invasion and metastasis of different cancers and their immune landscapes, showing that FAM83A over-regulation confers a very poor clinical outcome. Specifically, this strategic technique found that the number of naive B lymphocytes was inversely associated with the expression of FAM83A. Furthermore, a low density of immature B cells was statistically related to poor overall survival. In addition, using other critical methodologies—namely, LASSO, random forest, and BPNN algorithms—they identified that FAM83A drives immune cell infiltration. The investigation showed that FAM83A presents prognostic and predictive properties supported by machine-learning algorithms. Therefore, the unusual tumor permeation of naive B cells monitored by FAM83A may reveal robust biomarkers that preview the clinical behavior of patients with non–small cell lung cancer (10).

Using a similar technology, Liang et al. investigated the JMJD8 oncogene. They demonstrated an association with genome instability, whereas JMJD8 overexpression was related to overexpression of mismatch repair genes, stemness, a homologous repair gene signature, and a chromatin remodeling signature in more than nine cancers. Equally, a positive relationship was found between immune checkpoint CD276 and JMJD8. The next step was to use the same technology to validate JMJD8 as a marker of M2 macrophages and show its interaction with other cytotoxic immune cells, including CD8 T lymphocytes. Last but not least, drugs targeting JMJD8 were tracked and transformed in JMJD8 synthetic protein, resulting in a novel therapeutic approach for precision medicine.

Zhang et al., using next-generation sequencing and large-scale single-cell analyses, demonstrated the immune features of the CD147 marker and its impact on prognosis in different cancers. Notably, CD147 was overexpressed in almost all cancer types. CD147 expression modulates immune cell infiltration, immune checkpoints, and neoantigen rate in the tumor stroma. Furthermore, CD147 expression was detected on many cells in the tumor stroma, including malignant cells, histiocytes, lymphomononuclear cells, and cancer-associated fibroblasts. In addition, mIHC/IF analysis detected the co-localization pattern of CD147 and CD68, as well as CD163 macrophages, across the cancers included in the study. The immunotherapy response and sensitivity were evaluated after synthesizing small molecule drugs based on CD147 expression. The authors showed that CD147 detection was significantly associated with prognosis and immune infiltrates modulator across cancers. They suggested that silencing the CD147 gene may emerge as a promising therapeutic target in tumor immuno-oncology.

In summary, this special Research Topic collection encompasses the entire spectrum of current multiplexed image analysis technology from discovery findings (Hristu et al., Le Rochais et al., Hernandez et al.) to translational research (Rojas et al., Sundaram et al., 10, Liang et al., Zhang et al.) to proposed clinical implementation (Rojas et al.). The articles described in this Editorial characterize the applicability of multiplexed image technologies, discuss their advantages and disadvantages, and recognize that their application in clinical translational studies would help pathologists, oncologists, and research scientists in the field of cancer targeted therapy by helping to identify the best options for the application of these technologies, and engage stakeholders to standardize data validation techniques and practices.

## Author contributions

All authors listed have made a substantial, direct, and intellectual contribution to the work and approved it for publication.

## Acknowledgments

We thank the members of the Multiplex Immunofluorescence and Image Analysis Laboratory in the Department of Translational Molecular Pathology at MD Anderson Cancer Center, who contribute daily to quality analysis and interpretation of data from multiplexed image technologies. Editorial support was provided by Sunita Patterson from the Research Medical Library at MD Anderson Cancer Center.

## Conflict of interest

The authors declare that the research was conducted in the absence of any commercial or financial relationships that could be construed as a potential conflict of interest.

## Publisher’s note

All claims expressed in this article are solely those of the authors and do not necessarily represent those of their affiliated organizations, or those of the publisher, the editors and the reviewers. Any product that may be evaluated in this article, or claim that may be made by its manufacturer, is not guaranteed or endorsed by the publisher.
